# Aging, Cancer, and Inflammation: The Telomerase Connection

**DOI:** 10.3390/ijms25158542

**Published:** 2024-08-05

**Authors:** Virginia Boccardi, Luigi Marano

**Affiliations:** 1 Division of Gerontology and Geriatrics, Department of Medicine and Surgery, University of Perugia, 06132 Perugia, Italy; 2 Department of Medicine, Academy of Applied Medical and Social Sciences—AMiSNS: Akademia Medycznych I Spolecznych Nauk Stosowanych, 82-300 Elbląg, Poland; l.marano@amisns.edu.pl; 3 Department of General Surgery and Surgical Oncology, “Saint Wojciech” Hospital, “Nicolaus Copernicus” Health Center, 80-462 Gdańsk, Poland

**Keywords:** telomeres, aging, cancer, inflammation, cellular senescence, stem cells

## Abstract

Understanding the complex dynamics of telomere biology is important in the strong link between aging and cancer. Telomeres, the protective caps at the end of chromosomes, are central players in this connection. While their gradual shortening due to replication limits tumors expansion by triggering DNA repair mechanisms, it also promotes oncogenic changes within chromosomes, thus sustaining tumorigenesis. The enzyme telomerase, responsible for maintaining telomere length, emerges as a central player in this context. Its expression in cancer cells facilitates the preservation of telomeres, allowing them to circumvent the growth-limiting effects of short telomeres. Interestingly, the influence of telomerase extends beyond telomere maintenance, as evidenced by its involvement in promoting cell growth through alternative pathways. In this context, inflammation accelerates telomere shortening, resulting in telomere dysfunction, while telomere elements also play a role in modulating the inflammatory response. The recognition of this interplay has promoted the development of novel therapeutic approaches centered around telomerase inhibition. This review provides a comprehensive overview of the field, emphasizing recent progress in knowledge and the implications in understanding of cancer biology.

## 1. Introduction

Aging is a complex process that affects individuals on molecular, cellular, tissue, and systemic levels. It results from the cumulative effects of damage and the gradual decline of repair mechanisms, contributing to the onset of age-related diseases. As people age, they experience various physiological changes at the cellular and molecular levels, leading to an increased risk of cancer [1]. Common types of cancer in older adults include prostate cancer in men, breast cancer in women, and lung and colorectal cancers in both sexes [2]. The intersection of aging and cancer is a multifaceted issue arising from the interplay between aging processes and cancer development. In fact, aging is linked to a higher frequency of genetic mutations and genomic instability, all of which can predispose cells to malignant transformation [3]. Additionally, the efficiency of DNA repair mechanisms and immune surveillance declines with age, further increasing cancer risk. Chronic inflammation, characteristic of both aging and cancer, creates an environment conducive to cancer initiation, growth, and progression [4]. Addressing the complex relationship between cancer and aging requires a deep understanding of the underlying molecular and cellular processes and a personalized approach to cancer prevention, detection, and treatment. Despite many theories, no single explanation fully accounts for the aging process. Aging-related changes are often described by nine cellular and molecular hallmarks: genomic instability, telomere attrition, epigenetic alterations, loss of proteostasis, deregulated nutrient sensing, mitochondrial dysfunction, cellular senescence, stem cell exhaustion, and altered intercellular communication [3]. Recent reviews by López-Otín and colleagues [3,5] have expanded this list to include macroautophagy, dysbiosis, and chronic inflammation. Inflammation, an evolved defense mechanism to protect organisms from infection and injury, plays a crucial role in aging. Acute inflammation involves a sequence of responses to infection or injury that clears pathogens and initiates wound healing. In contrast, chronic inflammation, or “inflammaging”, is a pathological process closely linked to immune function decline [4,6]. Recent research has highlighted the significant impact of chronic inflammation on immune aging, showing a strong correlation between inflammation and telomere biology in various major health conditions, including cancer [7]. New evidence suggests that aging is driven by chronic inflammation, which depletes stem cells, disrupts cellular communication, and leads to telomere loss-telomeres being protective “caps” at chromosome ends [8]. To maintain telomere length and protect chromosomes from damage, highly proliferative cell types, such as hematopoietic progenitors and effector leukocytes, use the enzyme telomerase [9]. Telomerase activity is influenced by leukocyte proliferation, persistent inflammation, and the production of reactive oxygen species (ROS) [10]. While many studies have examined telomere length (TL) and telomerase activity as prognostic biomarkers in human cancer, this review focuses on potential interactions between inflammation and telomere biology in cancer development. Understanding the immune system’s interplay with telomerase activity could reveal new therapeutic targets for treating cancer and other age-related disorders [11].

## 2. Methodology

With this review, we conducted a literature search in PubMed, Medline, and Cochrane databases and concluded on 8 July 2024 to identify all articles published with the medical subject heading keywords “aging”, “cancer”, “older”, “telomere”, “telomerase”, “inflammation”, and “health”. The keywords were utilized in various combinations to maximize the retrieval of relevant articles, including studies ranging from bench and animal models to clinical trials. While priority was given to the most pertinent research, publications highlighting areas of interest were also included. This narrative review explores the complex interplay between aging and cancer with a particular focus on inflammation and telomerase connection. The review starts by introducing the telomere/telomerase biology. The journey continues with the exploration of telomerase connection in aging and cancer, followed by an assessment of inflammation and telomerase activity. The review concludes with a perspective on modulating the interaction between telomerase and inflammation.

## 3. The Telomere/Telomerase Biology: Implication in Aging and Cancer

Telomeres, which are repetitive DNA sequences (specifically TTAGGG in humans) at the ends of linear chromosomes, are crucial for genomic stability. During DNA replication, the end replication problem arises because the RNA primer at the 5′ end of the lagging strand provides a 3′ hydroxyl group. Once this primer is removed, DNA polymerase is unable to synthesize DNA to fill the resulting gap, leading to telomere shortening. Thus, telomeres, with each cell division or replication event, decrease in length, ultimately causing cells to enter a state of replicative senescence known as the “Hayflick limit”, where cells stop dividing. Due to this telomere attrition, telomeres are often referred to as a “biological clock” indicating cellular aging [12]. Although TL varies significantly among individuals, it consistently shortens with age and through each cell cycle, making it a recognized potential biomarker for aging [13]. When telomeres reach a critically short length, cells enter replicative senescence, leading to cell cycle arrest. This process is characterized by a stable cessation of cell proliferation, changes in gene expression, and a proinflammatory secretory phenotype [14]. The senescence-associated secretory phenotype (SASP) includes the release of various molecules, such as interleukins IL-1, IL-6, and IL-8, transforming growth factor (TGF)-β, and tumor necrosis factor (TNF)-α, which play significant roles in cellular signaling [15]. SASP is integral in modulating the immune response to senescent cells. Through the secretion of inflammatory cytokines, chemokines, and other immune modulators, senescent cells recruit immune cells to their vicinity, facilitating an immune response that targets the senescent cells for removal [15]. Importantly, the proteins secreted can function both in autocrine, affecting the cell that produced them, and paracrine, influencing neighboring cells, manners. External stressors can also induce premature senescence independent of TL, known as stress-induced premature senescence [16]. Oxidative agents, oncogenic signals, and ionizing radiation are common stimuli that can independently trigger premature cell senescence. Overall, cellular senescence plays a complex role in aging, development, and disease, acting both as a tumor-suppressive mechanism and as a contributor to tissue dysfunction and chronic inflammation [17,18].

Thus, telomere maintenance is a crucial aspect of cellular biology, influencing aging and cancer development. Three major factors are collectively involved in this context: the shelterin complex [19], the CTC1-STN1-TEN1 (CST) complex [20], and telomerase [21].

Shelterin, composed of six subunits (telomere repeat factors 1 and 2 (TRF1, TRF2), protection of telomeres 1 (POT1), repressor/activator protein 1 (RAP1), TRF1 and TRF2 interacting nuclear protein 2 (TIN2), and TPP1) plays a pivotal role in safeguarding chromosome ends and preventing the activation of the DNA damage response (DDR) [22,23]. The DDR pathways regulated by shelterin includes ATM (Ataxia Telangiectasia Mutated), ATR (Ataxia Telangiectasia and Rad3-related), Non-Homologous End Joining (NHEJ), Homologous Recombination (HR), and Alternative Lengthening of Telomeres (ALT) [24]. The ATM pathway, typically activated by double-strand breaks (DSBs), is essential for DNA repair, cell cycle regulation, and apoptosis. Shelterin, particularly through the TRF2 subunit, suppresses ATM activation at telomeres. TRF2 prevents the recognition of telomeres as DSBs, thereby blocking the recruitment of ATM and subsequent activation of downstream signaling, including phosphorylation of H2AX (γ-H2AX) and other checkpoint proteins [25]. The ATR pathway responds to a wide range of DNA damage, especially replication stress and single-stranded DNA (ssDNA). The POT1-TPP1 complex within shelterin is crucial in inhibiting ATR signaling at telomeres. POT1 binds to the single-stranded telomeric DNA overhang, preventing the recruitment of replication protein A (RPA), which is necessary for ATR activation. This, in turn, inhibits the phosphorylation of CHK1 and other ATR targets. NHEJ is a mechanism for repairing DSBs by directly ligating the broken DNA ends without a homologous template [26]. Shelterin inhibits NHEJ at telomeres via multiple mechanisms, with TRF2 preventing the recognition of telomeres as DSBs and blocking the recruitment of NHEJ factors such as Ku70/80 and DNA-PKcs [25]. The RAP1 component of shelterin also contributes to this suppression, ensuring that telomeres are not erroneously repaired through NHEJ, which could lead to chromosomal end-to-end fusions [27]. HR is a high-fidelity repair mechanism that uses a homologous sequence as a template. Although HR is generally suppressed at telomeres, in certain situations, such as telomere dysfunction, inappropriate HR activity may occur [28]. Shelterin, particularly through TRF2 and POT1, prevents the initiation of a DNA damage signal that would recruit HR machinery, thereby avoiding deleterious recombination events like sister chromatid exchanges or telomere–telomere recombination [22]. Finally, the ALT pathway is a recombination-based mechanism employed by some cancer cells to maintain telomere length in the absence of telomerase. Shelterin components, especially TRF1 and TRF2, are involved in repressing ALT activity. They achieve this by maintaining telomere integrity and preventing unscheduled recombination events that could result in telomere elongation via the ALT pathway [25,26,27]. Additionally, shelterin is essential for forming the T-loop structure, where the 3′ end of the telomere invades the double-stranded telomeric DNA, thereby preventing DDR activation [16,29].

The CTC1-STN1-TEN1 (CST) complex is a critical protein complex also involved in DNA replication and telomere maintenance [20]. It consists of three components: CTC1, which provides structural support and binds single-stranded DNA; STN1, which regulates DNA replication and telomere length; and TEN1, which stabilizes the complex and enhances DNA-binding affinity. The CST complex has several key functions, including telomere end protection, regulation of telomerase activity, rescue of stalled replication forks, and DNA replication stress response [20].

**Figure 1:**
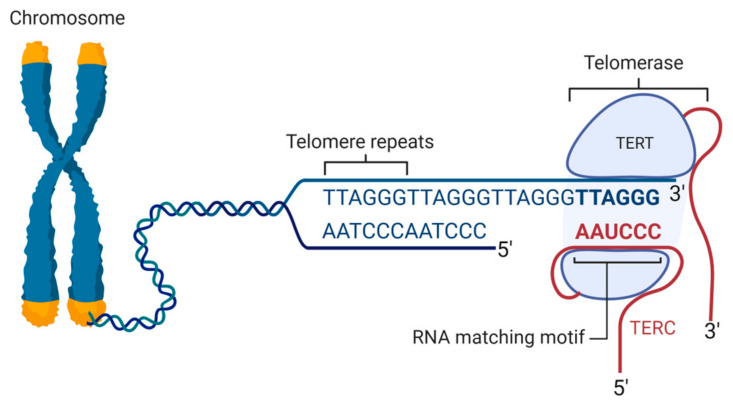
Human telomere structure and telomerase recruitment. Telomeric DNA consists of arrays of the TTAGGG telomeric repeat, forming a long region of double-stranded DNA terminating in the single-stranded G-rich overhang. Telomerase extends the length of shortened telomeres after DNA replication using TERC. TERC, telomerase RNA component; TERT, telomerase reverse transcriptase. Created with BioRender.com.

Telomerase is a ribonucleoprotein enzyme complex that plays a critical role in maintaining the length of telomeres as well as functions (Figure 1). The core components of telomerase include the telomerase reverse transcriptase (TERT) and the telomerase RNA component (TERC), which provides the template for telomere elongation. In detail, TERT is the catalytic protein subunit of telomerase, and functions as an RNA-dependent DNA polymerase to synthesize telomeric repeats [21]. It has reverse transcriptase activity, allowing it to synthesize DNA from an RNA template. The primary function of telomerase is to counteract telomere shortening that occurs during DNA replication. Telomerase helps balance genome stability and replication stress, reducing the latter which can be induced by oncogene activation, aneuploidy, or DNA-damaging agents. TERT’s ability to bind and repair stressed replication forks, coordinating DNA repair, underscores its role in maintaining genome integrity beyond just telomere elongation [30]. Interestingly, TERT also has several noncanonical roles that significantly impact aging and cellular function [31]. TERT has been found to localize to mitochondria, where it helps improve mitochondrial function and biogenesis. Studies have shown that TERT can enhance mitochondrial DNA repair and reduce mitochondrial ROS production, which is crucial for maintaining cellular energy production and reducing oxidative damage [32]. TERT’s involvement in the coordination of mitochondrial genome expression is crucial for maintaining cellular metabolism. It binds to mitochondrial DNA (mtDNA), protecting it from oxidative damage, and promotes the upregulation of antioxidant enzymes like superoxide dismutase, along with components of the electron transport chains production, and enhances mitochondrial function [33]. Interestingly, telomerase activation also alters the balance between catabolic and anabolic pathways for lipids and carbohydrates, which is linked to mitochondrial biogenesis [34]. TERT’s role in nuclear–mitochondrial communication helps cancer cells avoid DNA damage-induced apoptosis, highlighting its multifaceted functions in cancer cell adaptation and survival [30,35]. TERT directly regulates the transcription of numerous genes, including those involved in tumor growth, such as epidermal growth factor receptor (EGFR) and vascular endothelial growth factor (VEGF). TERT binds to the VEGF promoter and acts as an effector in VEGF signaling pathways. Inhibition of telomerase has been shown to reduce VEGF expression and angiogenesis [34]. Again, TERT interacts with signaling pathways such as Wnt/β-catenin and NF-κB, promoting cell proliferation and survival [36]. There is a reciprocal regulation between TERT and β-catenin, creating a feedback loop that sustains tumorigenic signaling. Upon nuclear import, TERT can bind NF-κB and regulate the expression of NF-κB-dependent genes that are crucial for tumor progression. TERT also interacts with the TGF-β-responsive, enhancing cancer cell dissemination and disease progression [34,35].

### 3.1. Telomerase and Aging: Insight from Age-Related Diseases and Progeroid Syndromes

Telomerase plays a crucial role in the relationship between telomeres and aging [13,37,38,39]. In most somatic cells, telomerase activity is low or absent, leading to progressive telomere shortening and eventual cell senescence. In contrast, stem cells, germ cells, and most cancer cells have higher telomerase activity, which helps maintain telomere length and allows these cells to divide indefinitely [13,38,39,40]. Telomerase deficiency and the resultant telomere shortening are associated with several age-related diseases, including idiopathic pulmonary fibrosis (IPF), aplastic anemia, and dyskeratosis congenita (DC) [41]. IPF is a chronic, progressive lung disease characterized by the scarring (fibrosis) of lung tissue. This fibrosis impairs the lungs’ ability to transfer oxygen into the bloodstream, leading to symptoms such as chronic cough and shortness of breath [42]. Telomerase deficiency contributes to IPF by causing premature shortening of telomeres in alveolar epithelial cells, which are critical for maintaining lung tissue integrity. The loss of regenerative capacity in these cells due to telomere dysfunction leads to increased cell death and fibrosis [41]. Patients with IPF often exhibit markedly shortened telomeres, and familial forms of the disease have been linked to mutations in genes encoding telomerase components. Aplastic anemia is a condition in which the bone marrow fails to produce enough blood cells, leading to pancytopenia—a deficiency of red cells, white cells, and platelets. This disease is strongly associated with telomerase deficiency, as the bone marrow’s hematopoietic stem cells (HSCs) are highly proliferative and, thus, susceptible to telomere attrition [43]. When telomeres become critically short, HSCs enter senescence or undergo apoptosis, resulting in reduced hematopoiesis and the clinical manifestations of anemia, susceptibility to infections, and bleeding disorders. Mutations in TERT and TERC genes are known to cause familial aplastic anemia, underscoring the critical role of telomerase in maintaining bone marrow function [43]. DC is a classic telomere biology disorder, often resulting from mutations in genes involved in telomere maintenance and other components of the telomerase complex or telomere-associated proteins. The hallmark of DC is extremely short telomeres in affected cells, leading to premature cellular senescence [44]. This manifests clinically as a broad spectrum of symptoms associated with defective tissue maintenance and regenerative capacity. Patients with DC may experience premature aging symptoms, including early graying, osteoporosis, and dental abnormalities, reflecting the systemic nature of telomere dysfunction [45].

Abnormalities in telomere structure and function are also seen in progeroid syndromes. Progeroid syndromes are a group of rare genetic disorders that mimic physiological aging at an accelerated rate. Werner syndrome (WRN) is a disorder that leads to premature aging, with symptoms such as hair loss, skin thinning, and various health complications. The WRN gene encodes the Werner syndrome ATP-dependent helicase (also known as WRN protein), which is crucial for maintaining genome stability. WRN is part of the RecQ helicase family, a group of enzymes involved in DNA repair, replication, recombination, and transcription. Werner syndrome results from mutations in the WRN gene, leading to telomere degradation and genomic instability [46]. Similarly, Bloom syndrome (BS) is a rare genetic disorder characterized by short stature, immune deficiency, and genomic instability due to mutations affecting helicase enzymes that regulate telomere length [47]. This increases the risk of cancer and other health issues. Nijmegen breakage syndrome (NBS), caused by mutations in the NBN gene, also results in genomic instability, increased cancer risk, and shortened telomeres [48]. Individuals with NBS often exhibit additional symptoms, including short stature, facial anomalies, recurrent infections, and developmental delays. Fanconi anemia (FA) typically manifests in childhood with various congenital abnormalities and developmental issues, such as short stature, microcephaly, and bone marrow failure, which predispose individuals to leukemia and squamous cell carcinoma [49]. Ataxia telangiectasia (A-T) begins in childhood and leads to progressive neurological decline, characterized by telangiectasia (prominent blood vessels in the eyes and skin) and increased sensitivity to radiation. A-T is associated with chromosomal abnormalities in T-cells and a higher risk of lymphoid tumors in childhood, along with increased susceptibility to gastrointestinal, endocrine, and breast cancers in adulthood [50]. Down syndrome (DS), resulting from an extra chromosome 21, causes developmental delays and intellectual disabilities. Individuals with DS have shortened telomeres, which contribute to accelerated aging and increased age-related health risks [51].

Interestingly, mice deficient in telomerase exhibit accelerated aging phenotypes, including hair greying, reduced wound healing, and early onset of osteoporosis [37,52]. On the contrary, studies using transgenic mice that overexpress TERT have shown extended lifespan and improved health span. These mice exhibited delayed onset of age-related diseases, enhanced tissue function, and reduced signs of aging [13]. Increased telomerase activity in mice has been linked to improved cardiovascular health, enhanced neurogenesis, and better metabolic function. These improvements suggest that telomerase can help maintain the overall health of various organ systems during aging. In humans, the relationship between telomerase activity and aging is more complex [53]. While maintaining optimal telomerase activity is associated with healthier aging and longer telomeres, excessive or uncontrolled activation poses a risk of cancer. This is because high telomerase activity allows cells to escape normal growth controls, leading to unchecked cell proliferation, a hallmark of cancer [53,54].

### 3.2. Telomerase and Cancer

Cancer cells are characterized by their ability to evade the normal regulatory mechanisms that control cell proliferation [55]. A critical barrier to unchecked cell division in healthy cells is the progressive shortening of telomeres with each cell division [35]. For normal cells to transform into cancerous cells, they must activate a telomere maintenance mechanism, typically through the upregulation of telomerase activity. Telomerase is crucial in the initiation and progression of cancer [34]. The persistent activity of telomerase and the resultant elongation of telomeres are essential for the unlimited proliferation of cancerous cells. In normal cells, telomere shortening triggers replicative senescence, a state where cells cease to divide. However, cancer cells circumvent this barrier by activating telomerase, which maintains telomere length and allows for continued cell division. This replicative immortality is achieved through the accumulation of genetic and epigenetic alterations that enable cancer cells to bypass senescence and transform into neoplastic cells [56]. During tumorigenesis, cancer cells acquire distinct characteristics, including sustained proliferative signaling, evasion of growth suppressors, enhanced invasion and metastasis, resistance to cell death, stimulation of angiogenesis, evasion of immune surveillance, immortalized replication, and reprogrammed cellular metabolism [34]. Emerging hallmarks such as phenotypic plasticity, nonmutational epigenetic changes, interactions with diverse microbiomes, and the presence of senescent cells further define the process of tumorigenesis [57]. Telomerase expression is significantly upregulated in many cancer types, in contrast to its minimal activity in normal somatic cells. This makes telomerase an attractive target for anticancer therapies. Inhibiting telomerase in cancer cells can induce apoptosis with minimal impact on normal cells [34,35]. Over 85% of human cancers maintain telomere length through telomerase activity, while the remainder use the ALT mechanism [13,58]. Inhibition of telomerase in cancer cell lines results in telomere shortening, reduced proliferation, and increased apoptosis, highlighting the enzyme’s critical role in sustaining cancer cell viability [13,34,35,37,55,56,57,58,59,60,61]. Cancer cells often reactivate telomerase to maintain telomere length, enabling unlimited proliferation. This reactivation can occur through various mechanisms, including genomic alterations, epigenetic modifications, and transcriptional activation. A common genomic alteration is mutations in TERT gene. These mutations create novel binding sites for transcription factors, such as ETS family factors, which enhance TERT transcription [62,63]. These gain-of-function mutations are prevalent in cancers like glioblastomas, melanomas, and bladder cancers [64]. Additionally, some cancers exhibit amplification of the TERT gene, resulting in increased mRNA and protein levels, as observed in hepatocellular carcinoma and neuroblastoma [65]. Structural chromosomal alterations, including translocations and inversions involving the TERT gene, can place it under the control of strong enhancers or promoters from other genes, leading to its upregulation. These rearrangements may also remove repressive elements or position TERT in a more transcriptionally active chromatin region [64]. In terms of epigenetic modifications, changes in the methylation status of the TERT promoter can significantly influence its activity. Hypomethylation of specific regions can lead to repression and activation of TERT expression, as seen in various cancers [66,67]. Furthermore, alterations in histone modifications, can result in a more open chromatin structure, facilitating transcription [68,69]. The recruitment of chromatin remodeling complexes that modify nucleosome positioning can also play a role in exposing the TERT promoter to transcription factors and the transcriptional machinery [67]. Regarding transcriptional activation, cancer cells often upregulate transcription factors that directly or indirectly increase TERT expression. Factors such as c-Myc and NF-κB have been shown to bind the TERT promoter and enhance its transcription. Mutations or overexpression of these factors further contribute to telomerase reactivation [70,71]. Oncogenic signaling pathways, including PI3K/AKT, MAPK/ERK, and WNT/β-catenin, can also activate TERT transcription by converging on transcription factors or coactivators [72]. Additionally, alternative splicing of TERT transcripts can produce isoforms with varying activity. Long noncoding RNAs (lncRNAs) and microRNAs (miRNAs) can regulate TERT expression post-transcriptionally, with some miRNAs downregulating repressors of TERT or directly enhancing its expression [73].

## 4. Chronic Inflammation and Telomerase Activity

The complex relationship between inflammation and telomerase activity significantly impacts cellular aging and cancer progression [74]. Chronic inflammation is a prolonged and persistent inflammatory response characterized by the continuous activation of the immune system and the sustained presence of proinflammatory cytokines and other mediators [75]. Unlike acute inflammation, which is a short-term and immediate response to injury or infection aimed at restoring tissue homeostasis, chronic inflammation can last for months or even years. It often arises from unresolved acute inflammation, persistent infections, prolonged exposure to irritants, or autoimmune disorders, where the immune system erroneously targets the body’s own tissues [76]. In detail, chronic inflammation persists over long periods, often without a clear resolution. It involves the persistent activation of various immune cells, including macrophages, lymphocytes, and plasma cells. Elevated levels of proinflammatory cytokines such as IL-6, TNF-α, and C-reactive protein (CRP) are commonly observed. Chronic inflammation can lead to tissue remodeling, fibrosis, and the formation of granulomas, which are aggregates of immune cells formed to wall off foreign substances that the body cannot eliminate [39,77]. Chronic inflammation is a significant underlying factor in various chronic diseases and conditions, impacting overall health and aging processes. It is closely linked to the development of numerous age-related diseases, including cardiovascular diseases, diabetes, neurodegenerative disorders (such as Alzheimer’s disease), and certain cancers. The continuous inflammatory response can cause damage to tissues and organs, contributing to disease progression. Chronic inflammation accelerates cellular aging through mechanisms such as increased oxidative stress, DNA damage, and the promotion of cellular senescence [38,78]. Senescent cells, in turn, secrete proinflammatory factors, perpetuating a cycle of inflammation and tissue damage. Chronic inflammation can interfere with metabolic processes, leading to insulin resistance, obesity, and metabolic syndrome. These conditions further exacerbate inflammatory responses, creating a feedback loop that can be challenging to break. Persistent inflammation can lead to immune system dysregulation, making the body more susceptible to infections and reducing its ability to respond effectively to new threats [79]. This dysregulation is particularly concerning in older populations, who already have a naturally declining immune function.

### The Impact of Chronic Inflammation on Telomerase Activity

Chronic inflammation significantly impacts telomere dynamics, influencing cellular aging and health outcomes. The following are the main linking mechanisms:

- Telomerase inhibition: Proinflammatory cytokines such as IL-6 and TNF-α can inhibit telomerase activity, particularly the catalytic subunit TERT. This inhibition accelerates telomere shortening, leading to premature cellular senescence [80].
- Oxidative stress: Chronic inflammation increases oxidative stress by generating ROS. ROS directly damage telomeres, further contributing to their attrition and the aging process [33].
- Senescence and SASP: Cells with critically short telomeres often enter a state of senescence, characterized by the secretion of SASP. SASP factors perpetuate local and systemic inflammation, creating a feedback loop that exacerbates telomere dysfunction and cellular aging [81].
- Mitochondrial dysfunction: The telomere–mitochondrial axis is crucial in understanding aging processes. Telomere shortening disrupts mitochondrial function, leading to increased ROS production and further oxidative damage. Dysfunctional mitochondria exacerbate cellular aging and promote the inflammatory response [82,83].

In detail, persistent inflammatory signals inhibit the expression and function of the catalytic subunit TERT, accelerating telomere shortening and promoting cellular senescence [84]. Studies from Blackburn et al. [75] and Deo et al. [85] have shown that elevated levels of these cytokines are closely associated with accelerated telomere shortening. Chronic inflammation-induced oxidative stress generates ROS that directly damage telomeres, exacerbating their shortening. Jurk et al. [77] demonstrated in murine models that chronic inflammation, induced via the knockout of the nfkb1 subunit of the NF-κB transcription factor, exacerbates telomere dysfunction and cellular senescence. This feedback loop involving NF-κB and ROS accelerates aging processes and diminishes tissue regeneration in organs like the liver and gut. While chronic inflammation generally inhibits telomerase activity, acute inflammation can transiently activate telomerase as a protective response to preserve telomere length and cellular function. This transient activation helps mitigate immediate damage and maintain cellular integrity. However, the protective effects of acute inflammation are often outweighed by the detrimental impacts of chronic inflammation, leading to overall telomere attrition and accelerated aging. The interplay between telomere shortening and mitochondrial dysfunction is a critical factor in cellular aging. Telomere shortening disrupts normal cellular function and contributes to increased ROS production, exacerbating mitochondrial dysfunction and cellular aging [78]. Mitochondria, as both producers and targets of ROS, accumulate damage, leading to dysfunctional mitochondrial DNA (mtDNA) and further impairing mitochondrial function [74]. Dysfunctional telomeres can activate p53, inhibiting the transcription of peroxisome proliferator-activated receptor gamma coactivator 1-alpha (PGC-1α), a key regulator of mitochondrial biogenesis and function. Reduced PGC-1α levels result in mitochondrial dysfunction and increased ROS production [38]. Cells with critically short telomeres often undergo senescence, accompanied by a proinflammatory SASP, which further exacerbates mitochondrial dysfunction and oxidative stress [39,79].

## 5. Therapeutic Strategies: A Comprehensive Overview of the Key Studies and Findings

Therapeutic strategies targeting the interplay between telomerase and inflammation hold promise for treating a range of age-related diseases and cancer [13]. By enhancing telomerase activity, it may be possible to preserve telomere length, reduce cellular senescence, and promote tissue regeneration, thereby alleviating symptoms and progression of diseases associated with aging, such as cardiovascular diseases, neurodegenerative disorders, and diabetes [13]. Conversely, in cancer, where telomerase is often upregulated to support uncontrolled cell proliferation, telomerase inhibitors can be employed to limit tumor growth and enhance the effectiveness of existing treatments [34]. Additionally, modulating inflammatory pathways that influence telomerase activity presents another therapeutic avenue. For instance, anti-inflammatory agents that reduce oxidative stress and cytokine production could mitigate telomere attrition and improve cellular health in chronic inflammatory conditions [86]. Combining telomerase activators with antioxidants or anti-inflammatory drugs might offer synergistic benefits, enhancing cell repair mechanisms while controlling harmful inflammation [87]. Looking forward, personalized medicine approaches that consider individual variations in telomerase activity and inflammatory responses could optimize these therapeutic strategies, providing more effective and targeted treatments for patients with age-related diseases and cancer. The development of such therapies could significantly improve health span and quality of life, marking a substantial advancement in the management of these conditions.

The modulation of telomerase activity through various compounds and genetic modifications has shown promising results in both preclinical and clinical studies [88,89,90,91,92,93,94]. Telomerase modulators as therapeutic agents is a rapidly evolving field. While preclinical studies have shown promising results, the translation to clinical success has been challenging. Table 1 provides a comprehensive overview of the key studies and findings in these areas.

Imetelstat (GRN163L) is a telomerase inhibitor that has demonstrated its ability to inhibit tumor growth and metastasis in mouse models of various cancers, including breast and lung cancer [95]. In humans, Phase II clinical trials have shown partial responses and disease stabilization in patients with myelofibrosis and certain solid tumors, indicating its potential therapeutic value [96]. BIBR1532 is a telomerase inhibitor has been shown to suppress tumor growth in xenograft models of glioblastoma and leukemia [94]. However, evidence in humans is currently lacking, as BIBR1532 remains in the preclinical study phase with no clinical trials conducted yet [97]. 6-thio-2′-deoxyguanosine (6-thio-dG), as a telomerase substrate that induces telomere dysfunction, has reduced tumor growth and improved survival in mouse models of glioblastoma and melanoma [93]. While preclinical studies are promising, clinical trials in humans have not yet been initiated [98]. Curcumin, a natural compound, inhibits telomerase activity [99]. It has been shown to decrease telomerase activity and tumor growth in mouse models of prostate cancer. Although limited clinical studies suggest potential benefits for humans, more extensive research is necessary to confirm its efficacy [92,97,99]. Resveratrol is another natural compound that modulates telomerase and telomeres [100]. In animal models, it has been found to extend lifespan and reduce tumor incidence, though its effects on telomerase activity are mixed. Some clinical trials indicate potential anticancer effects, but data on telomerase modulation in humans are still limited [91,100,101]. RHPS4 is a G-quadruplex stabilizer that inhibits telomerase and has been shown to reduce tumor growth and cause telomere shortening in mouse models of melanoma and glioblastoma [90]. Currently, RHPS4 is in the preclinical study phase, showing potential for future clinical development [80]. TA-65, a telomerase activator derived from Astragalus membranaceus, has improved health span and telomere length in aged mice. However, data on its effects on cancer are mixed [13]. Some human studies suggest telomere lengthening and improved markers of aging, but its impact on cancer remains limited and controversial [13]. Genetic modifications to increase telomerase expression through TERT promoter mutations have been shown to extend lifespan and delay cancer onset in some transgenic mouse models, although they also increase cancer risk in others [102]. In humans, these mutations are observed in various cancers, with some patients showing increased telomerase activity contributing to tumor progression. GRNVAC1 is a telomerase-based dendritic cell vaccine that has induced immune responses and reduced tumor burden in mouse models of prostate cancer [103]. In Phase II clinical trials, it showed immune activation and potential clinical benefits in patients with acute myeloid leukemia [104]. Cycloastragenol, another extract from Astragalus, acts as a telomerase activator. It has been shown to improve telomere length and reduce oxidative stress in aged mice [105]. Some small human studies indicate telomere lengthening, but more research is needed to determine its effects on cancer [89,106,107]. In conclusion, various telomerase modulators, including inhibitors, activators, and genetic modifications, have shown potential in preclinical and clinical studies. These modulators offer promising avenues for cancer therapy and age-related disease management, but further research is needed to fully understand their mechanisms and optimize their clinical application. The relationship between inflammation and telomerase is complex and bidirectional, significantly influencing cellular aging and cancer progression. Chronic inflammation inhibits telomerase activity through oxidative stress and downregulation of TERT, leading to telomere shortening and cellular senescence. Conversely, telomerase can modulate inflammatory responses and enhance mitochondrial function, reducing inflammation-induced damage. Understanding and modulating this interplay is crucial for developing therapeutic strategies to treat age-related diseases and cancer, ultimately promoting healthy aging and improved quality of life [63].

## 6. Conclusions and Perspectives

**Figure 2:**
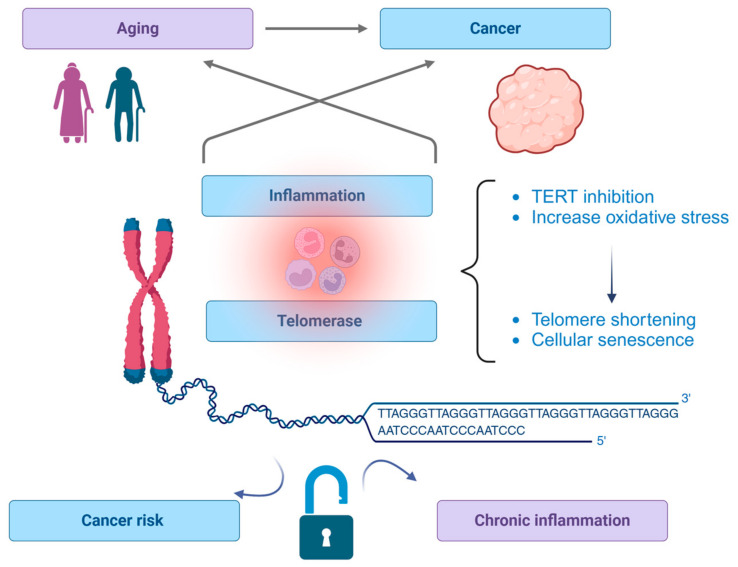
Telomerase: The link between aging, cancer, and inflammation. This figure shows the complex interrelationships between aging, cancer, inflammation, and telomerase. It underscores the importance of telomerase in maintaining cellular integrity and its potential role in mediating both cancer risk and chronic inflammatory conditions. This visualization aids in understanding how these biological processes are interconnected and highlights the significance of telomerase as a therapeutic target in addressing age-related diseases and cancer. Created with BioRender.com.

In conclusion, Figure 2 illustrates the interconnected relationships between aging, cancer, inflammation, and telomerase activity. It shows how these processes influence each other, contributing to cancer risk and chronic inflammation. The central role of telomerase and its relationship with telomeres is emphasized. In the present day, telomerase is recognized for its critical role in both cellular aging and cancer progression. Recent research suggests that interventions aimed at preserving telomere length through lifestyle modifications, such as exercise and a balanced diet, can promote healthy aging and reduce the risk of age-associated diseases [88,108]. In cancer, the reactivation of telomerase is a crucial step for the immortalization of cells [80]. Telomerase not only supports the unlimited growth of cancer cells by elongating telomeres but also contributes to cancer progression through its noncanonical roles [62]. The regulation of telomerase expression, assembly, and activity is complex, occurring at multiple levels of gene expression. It is influenced by various intracellular and environmental stimuli, many of which are also involved in malignant transformation and tumor progression [34]. Key components of telomerase, including TERT and TERC, play vital roles in these regulatory processes. TERT, for instance, can translocate to mitochondria under oxidative stress, where it enhances mitochondrial function and helps maintain cellular redox balance [35]. Animal models have been instrumental in elucidating the roles of telomerase in aging and cancer. Transgenic mice that overexpress TERT show extended lifespans and delayed onset of age-related diseases, while telomerase-deficient mice exhibit premature aging and reduced tissue regeneration [30]. In cancer models, telomerase inhibition leads to telomere shortening, reduced tumor growth, and increased apoptosis, underscoring its potential as a therapeutic target [63]. Future research aims to further explore the mechanisms by which telomerase and telomere dynamics influence aging and related diseases. Potential therapeutic interventions include the development of small molecule activators of telomerase, gene therapy approaches, and lifestyle modifications designed to enhance telomere maintenance [103]. The goal is to promote healthy aging and extend health span while minimizing the risk of oncogenesis. As we move forward, interdisciplinary collaboration and the integration of cutting-edge technologies, such as genomics, bioinformatics, and nanotechnology, will be crucial.

## Figures and Tables

**Table 1:** The main current state of research and key studies on telomerase modulators in cancer; different approaches and levels of evidence from animal and human studies.

Modulator	Mechanism	Evidence in Animals	Evidence in Humans
**Imetelstat (GRN163L)**	Telomerase inhibitor.	Inhibited tumor growth and metastasis in mouse models of various cancers, including breast and lung cancer.	Phase II clinical trials showed partial responses and disease stabilization in patients with myelofibrosis and certain solid tumors.
**BIBR1532**	Telomerase inhibitor.	Suppressed tumor growth in xenograft models of glioblastoma and leukemia.	Preclinical studies only, no clinical trials yet.
**6-thio-2′-deoxyguanosine (6-thio-dG)**	Telomerase substrate that induces telomere dysfunction.	Reduced tumor growth and improved survival in mouse models of glioblastoma and melanoma.	Preclinical studies only, showing promise for future clinical trials.
**Curcumin**	Natural compound, inhibits telomerase activity.	Decreased telomerase activity and tumor growth in mouse models of prostate cancer.	Limited clinical studies suggest potential benefits, but more research is needed.
**Resveratrol**	Natural compound, modulates telomerase and telomeres.	Extended lifespan and reduced tumor incidence in animal models; effects on telomerase activity are mixed.	Some clinical trials indicate potential anticancer effects, though data on telomerase modulation in humans is limited.
**RHPS4**	G-quadruplex stabilizer, inhibits telomerase.	Reduced tumor growth and telomere shortening in mouse models of melanoma and glioblastoma.	Preclinical studies only, showing potential for clinical development.
**TA-65**	Telomerase activator, derived from Astragalus membranaceus.	Improved health span and telomere length in aged mice; data on cancer effects are mixed.	Some human studies suggest telomere lengthening and improved markers of aging, but data on cancer effects is limited and controversial.
**Tert promoter mutations**	Genetic modifications to increase telomerase expression.	Extended lifespan and delayed cancer onset in some transgenic mouse models; increased cancer risk in others.	Observed in various cancers; some patients show increased telomerase activity, contributing to tumor progression.
**GRNVAC1**	Telomerase-based dendritic cell vaccine.	Induced immune response and reduced tumor burden in mouse models of prostate cancer.	Phase II clinical trials showed immune activation and potential clinical benefits in patients with acute myeloid leukemia.
**Cycloastragenol**	Telomerase activator, another Astragalus extract.	Improved telomere length and reduced oxidative stress in aged mice; effects on cancer are unclear.	Some small human studies indicate telomere lengthening, but more research is needed to determine cancer-related effects.

## Data Availability

Not applicable.
